# An equivalence test between features lists, based on the Sorensen–Dice index and the joint frequencies of GO term enrichment

**DOI:** 10.1186/s12859-022-04739-2

**Published:** 2022-05-31

**Authors:** Pablo Flores, Miquel Salicrú, Alex Sánchez-Pla, Jordi Ocaña

**Affiliations:** 1 Escuela Superior Politécnica de Chimborazo (ESPOCH), Research Group in Data Science CIDED, Panamericana Sur Km 1 1/2, Riobamba, Ecuador; 2 Department of Genetics, Microbiology and Statistics, Statistics Section, Universitat de Barcelona, Av. Diagonal 643, 08028 Barcelona, Spain; 3 Statistics and Bioinformatics Unit, Vall d’Hebron Institute of Research (VHIR), Vall d’Hebron 119-129, 08035, Barcelona, Spain; 4 Department of Statistics and Operational Research, Faculty of Mathematics and Statistics, Universitat Politècnica de Catalunya, Barcelona, Spain

**Keywords:** Delta method, Bootstrap, Simulation, Type I error, Irrelevance of dissimilarity, Gene lists

## Abstract

**Background:**

In integrative bioinformatic analyses, it is of great interest to stablish the equivalence between gene or (more in general) feature lists, up to a given level and in terms of their annotations in the Gene Ontology. The aim of this article is to present an equivalence test based on the proportion of GO terms which are declared as enriched in both lists simultaneously.

**Results:**

On the basis of these data, the dissimilarity between gene lists is measured by means of the Sorensen–Dice index. We present two flavours of the same test: One of them based on the asymptotic normality of the test statistic and the other based on the bootstrap method.

**Conclusions:**

The accuracy of these tests is studied by means of simulation and their possible interest is illustrated by using them over two real datasets: A collection of gene lists related to cancer and a collection of gene lists related to kidney rejection after transplantation.

**Supplementary Information:**

The online version contains supplementary material available at 10.1186/s12859-022-04739-2.

## Background

Omics technologies have revolutionized 21st century biology and medicine [1] by making it possible to conduct massive studies of biological characteristics. The possibility they offer to simultaneously study the behavior or the changes experienced in all the genes of an organism, or the proteins or metabolites, has allowed to tackle new approaches, for example, to discover biomarkers of diseases, classify individuals based on these traits or simply to better understand biological processes, adopting a systems biology approach, which requires information on all components of the system [2].

While it is true that there are many different types of omic studies, a common characteristic of many of them is that they often result in one or more lists of “characteristics”, for example, genes that are differentially expressed between two conditions, proteins that interact physically in a certain tissue, metabolites associated with a given phenotype (“metabotypes”), and so on [3]. These lists, which will be the object of our study, will be described, from now on, as “features lists”.

Feature list analysis has had a curious history in the last two decades. Scientists quickly realized that a list of genes (this all started with genes) had to contain hidden or implicit information that could be useful for the biological interpretation of the results of the experiment that generated the list. From here the most commonly used functional analysis methods were born [4] such as Over-representation Analysis [5], and the GSEA method [6], which are based on the distribution of the annotations of the selected genes among different categories (“gene sets”) that represent, for example, different biological processes. Dozens of variations of these methods have been developed and implemented in a miriad of R packages and web tools. The clusterProfiler R package [7] is probably the state of the art of these tools.

Given the interest in studying lists of individual genes, one might hope that a reasonable “next step” would have been the development of methods to somehow compare lists of characteristics, which would be equivalent to comparing the experiments or studies that generated them. However, there was no massive development of approaches for comparing gene lists. In fact, in the almost 20 years that have passed since the advent of Gene Enrichment Analysis, only a few approaches for comparing feature lists have been suggested, such as VennPainter [8], or ToppCluster [9] most of them descriptive, and only a few of them with statistical justification to support the comparison. [10] provide an updated comparison of such tools.

One of these approaches has been the goProfiles method, developed by the authors [10–12]. This evolved from just being able to make comparisons between two lists of genes, to extending these to equivalence tests, first between two lists and finally to a collection of lists. The Bioconductor package, goProfiles, ( [13]) available since 2008, implements this method and has evolved since then to incorporate these improvements.

### An inferential approach for the comparison of features lists

Our previous papers on the “goProfiles” methodology, [10, 11] proposed some inferential approaches for comparing features lists. Its core idea is to compare two features lists on the basis of their observed “annotation profiles” in an ontology from Gene Ontology GO [14, 15]. Specifically, two features lists are compared through their vectors, ${\hat{P}}$ and ${\hat{Q}}$, of annotation frequencies in the terms of a given set of selected GO categories, like those in a given GO level. Both gene lists may be considered just as samples of the genes that could be selected in the respective experiments that generated them. If *d* is a dissimilarity index, $d({\hat{P}},{\hat{Q}})$ may be understood as a measure of the difference in their biological meaning. In the before cited papers, the chosen dissimilarity index *d* was the squared Euclidean distance $d^2_E$, but the results can be extended to other measures of dissimilarity. If *P* and *Q* stand for the corresponding population profiles, rejecting the null hypothesis in $H_0:d^2_E(P, Q)=0$ vs $H_1:d^2_E(P, Q)>0$ provides some evidence on a true difference in their biological meaning, i.e., on the hypothesis of a non-null dissimilarity. But “statistically significant” should not be confused with “biologically important”. This fact, jointly with the adoption of a data integrative approach, led to consider an equivalence testing point of view [16], $H_0:d^2_E(P, Q)\ge \Delta$ vs $H_1:d^2_E(P, Q) < \Delta$, considered in [12]. Rejecting $H_0$ provides evidence to conclude irrelevant dissimilarity (not necessarily null) between profiles, up to a threshold $\Delta$.

During the reviewing process of the above mentioned paper, an interesting point emerged: It was stressed the fact that, in the goProfiles approach, all GO terms under consideration are treated equitatively a priory. The importance of a given GO term is just reflected by how many genes in the list are annotated in it. On the other hand, provided the central role that the status of being an “enriched term” (e.g., [6]) plays in the GO-based analysis of gene lists, one may think in the possibility of measuring the dissimilarity between two gene lists as a decreasing function of how many enriched terms they share in common, among a given set of GO terms. For this purpose, we used the Sorensen–Dice index [17] as an adequate way (possibly among others) to measure the dissimilarity between two gene lists.

The present paper presents a methodology devoted to compare gene lists on the basis of this idea. The next section outlines the main theoretical and simulation results which sustain this approach. Its main goals are (i) To motivate the use of the Sorensen–Dice index, (ii) To present some asymptotic results on the sampling distribution of this index, (iii) To present an equivalence test for dissimilarity negligibility, discussing also the rationale of possible numerical specifications of the equivalence threshold, (iv) To consider the problem of simultaneously comparing more than two gene lists and, finally, (v) To study the degree of accuracy of the asymptotic theory by means of simulations and to introduce a bootstrap approach which improves this accuracy. The third section presents two case-studies based on real data: One of them is a comparative study of some gene lists related to cancer (allOnco gene lists) and the other corresponds to a study of gene lists related to rejection problems in kidney transplants, based on the Pathogenesis-based transcripts sets (PBTs). These examples also serve to compare the results provided by the present method with those obtained with another inferential method, goProfiles. The paper ends with a discussion on the pros and cons of this approach, comparing it with the before cited inferential method goProfiles.

## Methods

### Sorensen–Dice index

As has been previously outlined, the method consists in projecting the gene lists to be compared into a given set of GO terms. The dissimilarity between the gene lists is measured in terms of how many of these GO terms are enriched in both lists, how many are enriched in the first list but not in the second, etc. In other words, the degree of coincidence, or not, of both lists in terms of enrichment in the reference set of GO terms. It should be clear that the data being analysed/compared are gene lists, and the GO plays the role of a frame of reference for this analysis.

Given a previously fixed set of *n* GO terms (like all terms in a specific GO level) and an enrichment testing method (like the Fisher’s test with Bonferroni correction for a significance level like 0.05), the incidence of enriched terms in two gene lists may be cross-tabulated as in Table 1, where $n_{00}$ stands for how many GO terms are non-enriched in both lists, $n_{01}$ for the terms non-enriched in the first list but enriched in the second one, $n_{10}$ the reverse, enriched in the first list but not in the second, and finally $n_{11}$ for those enriched in both lists.

In terms of enrichment incidence, one may think in the dissimilarity between two gene lists as a decreasing function of the degree of coincidence in enrichment $n_{11}$, i.e., how many GO terms were declared as enriched in both lists. One may think in many admissible ways to express this dissimilarity. In our opinion, a compelling condition to choose a measure is that it should not include the double negatives $n_{00}$ in its computation. Note that this frequency may be inflated artificially if the total number *n* of terms to be considered grows, e.g., going deeper in the GO (terms correspond to more and more specific concepts) and considering all terms in each level.

Without excluding other possibilities, in this paper we opted for the Sorensen–Dice index [17]. Adapted to the context of the present scope of applications, and expressing it as a dissimilarity, a definition close to the original idea is:

1 $$\begin{aligned} d_S = d_S(p) = d_S({p}_{11}, {p}_{01}, {p}_{10}) = 1 - \frac{2{p}_{11}}{2{p}_{11} + {p}_{10} + {p}_{01}} \end{aligned}$$

where $$p = \left( p_{11}, p_{01}, p_{10}\right)$$ and:

$$\begin{aligned}\begin{array}{l} {p_{11}} = \Pr \left\{ \text{``GO term enriched in both lists''} \right\} \\ {p_{01}} = \Pr \left\{ \text{``GO term only enriched in the second list''} \right\} \\ {p_{10}} = \Pr \left\{ \text{``GO term only enriched in the first list''} \right\} \\ {p_{00}} = \Pr \left\{ \text{``GO term not enriched in the first list, nor in the second''} \right\} \\ \quad \quad = 1 - \left( {{p_{11}} + {p_{01}} + {p_{10}}} \right) \end{array}\end{aligned}$$

Given data like those outlined in Table 1, this dissimilarity can be estimated as:

2 $$\begin{aligned}  {\hat{d}}_S = d_S({\hat{p}}) = d_S({{\hat{p}}}_{11}, {{\hat{p}}}_{01}, {{\hat{p}}}_{10}) & = 1 - \frac{2{{\hat{p}}}_{11}}{2{{\hat{p}}}_{11} + {{\hat{p}}}_{10} + {{\hat{p}}}_{01}} \\ &= 1 - \frac{2{n}_{11}}{2{n}_{11} + {n}_{10} + {n}_{01}}  \end{aligned}$$

with $${\hat{p}}_{ij} = n_{ij}/n$$.

The Sorensen–Dice index takes values in the [0,1] interval. If this value approaches 0, it means that there is a predominance of positive dependence on the enrichment degree of both lists $L_1, L_2$, which seems reasonable to identify with biological similarity between them. On the other hand, if the index is close to 1, finding a GO term which is enriched in both lists is a rare event, also reasonably identifiable with great biological dissimilarity.

This index is widely used in other research areas like ecological studies, where typically $p_{11}$ corresponds to the proportion of species common to two biological communities, $p_{10}$ to the proportion of species present in the first community but not in the second and $p_{01}$ to those present in the second but not in the first one. Again, it therefore seems inappropriate to inflate the total with (possibly many candidate) species not present in any of these communities, the double negatives.

**Table 1:** Contingency table for frequencies of enriched and non enriched GO terms in two gene lists $L_1$ and $L_2$

	Enriched in ${\mathbf{L}}_{\boldsymbol{2}}$	Non enriched in ${\mathbf{L}}_{\boldsymbol{2}}$	
Enriched in $L_1$	$n_{11}$	$n_{10}$	$n_{1.}$
Non enriched in $L_1$	$n_{01}$	$n_{00}$	$n_{0.}$
	$n_{.1}$	$n_{.0}$	*n*

### Asymptotic theory for Sorensen dissimilarity

As has been mentioned, the Sorensen–Dice index has been used in other areas, principally in mathematical ecology. To our knowledge, these applications are mainly descriptive, with the exception of [18] where the statistical error associated to their values is measured by means of the bootstrap variance. Here we take a different approach based on the delta method [19]. According to it (see Additional file 1: Mathematical Details in Appendix), $\sqrt{n} \left( {{d_S}\left( {{\hat{p}} } \right) - {d_S}\left( p \right) } \right)$ is asymptotically normal with variance:

3 $$\begin{aligned} \sigma _S^2 = \frac{{4{p _{11}}\left( {{p _{01}} + {p _{10}}} \right) \left( {{p _{11}} + {p _{01}} + {p _{10}}} \right) }}{{{{\left( {2{p _{11}} + {p _{01}} + {p _{10}}} \right) }^4}}}, \end{aligned}$$

which can be estimated as:

4 $$\begin{aligned} {{\widehat{\sigma }} _S^2} = \frac{{4{{\hat{p}} _{11}}\left( {{{\hat{p}} _{01}} + {{\hat{p}} _{10}}} \right) \left( {{{\hat{p}} _{11}} + {{\hat{p}} _{01}} + {{\hat{p}} _{10}}} \right) }}{{{{\left( {2{{\hat{p}} _{11}} + {{\hat{p}} _{01}} + {{\hat{p}} _{10}}} \right) }^4}}}. \end{aligned}$$

In consequence, a two-sided confidence interval with confidence level $1 - \alpha$ for $d_S(p)$ is given by:

5 $$\begin{aligned} d_S({\widehat{p}}) \pm z_{\alpha /2} \frac{{\widehat{\sigma }} _S}{\sqrt{n}} = d_S({\widehat{p}}) \pm z_{\alpha /2} \frac{2 \sqrt{{{{\hat{p}} _{11}}\left( {{{\hat{p}} _{01}} + {{\hat{p}} _{10}}} \right) \left( {{{\hat{p}} _{11}} + {{\hat{p}} _{01}} + {{\hat{p}} _{10}}} \right) }}}{{{{\left( {2{{\hat{p}} _{11}} + {{\hat{p}} _{01}} + {{\hat{p}} _{10}}} \right) }^2 \sqrt{n}}}} \end{aligned}$$

and, more interestingly for the objectives of this paper, the upper limit $d_u$ of a one-sided confidence interval $[0,d_u]$ of level $1 - \alpha$ is given by:

6 $$\begin{aligned} d_S({\widehat{p}}) - z_{\alpha } \frac{{\widehat{\sigma }} _S}{\sqrt{n}} = d_S({\widehat{p}}) - z_{\alpha } \frac{2 \sqrt{{{{\hat{p}} _{11}}\left( {{{\hat{p}} _{01}} + {{\hat{p}} _{10}}} \right) \left( {{{\hat{p}} _{11}} + {{\hat{p}} _{01}} + {{\hat{p}} _{10}}} \right) }}}{{{{\left( {2{{\hat{p}} _{11}} + {{\hat{p}} _{01}} + {{\hat{p}} _{10}}} \right) }^2 \sqrt{n}}}}. \end{aligned}$$

In the above  formulae $z_{\alpha /2}$ and $z_{\alpha }$ correspond to the $\alpha / 2$ and $\alpha$ quantiles of the standard normal distribution, respectively.

### Equivalence test

As is outlined in the background section, an equivalence test (i.e., a test of dissimilarity irrelevance between two gene lists) based on the Sorensen–Dice dissimilarity and the contingency tables of mutual enrichment may be formulated in the following terms:

7 $$\begin{aligned} \begin{aligned} H_0&: d_S(p) \ge d_0 \\ H_1&: d_S(p) < d_0, \end{aligned} \end{aligned}$$

where $d_0$ stands for a given equivalence limit or, in other words, a limit of dissimilarity irrelevance.

According to the interval inclusion principle (e.g., [16]), to reject the null hypothesis in (7) if the one-sided $1 - \alpha$ confidence interval $[0, d_u]$ defined in (6) is completely inside the parametric region of $H_1$ (i.e., if $d_u < d_0$), defines a test with type I error probability at most $\alpha$. This is a way to stablish biological equivalence between lists $L_1$ and $L_2$ up to a level $d_0$. Rejecting $H_0$ corresponds to establishing the irrelevance (up to a level $d_0$) of the dissimilarity between both lists, for a dissimilarity which is based on the degree of GO terms coincidence in enrichment.

The above decision criterion may be also reformulated in terms of *p*-values: $H_0$ will be rejected if $p(d_0) \le \alpha$, with

8 $$\begin{aligned} p(d_0) = \Phi \left( \frac{\sqrt{n}(d_S({\widehat{p}}_{11}) - d_0)}{{\widehat{\sigma }}_{S}}\right) , \end{aligned}$$

where $\Phi$ stands for the standard normal cumulative distribution function and $p(d_0)$ for the p–value when the equivalence limit is $d_0$.

### Equivalence threshold

Although the equivalence limit $d_0$ will always be an arbitrary value, it may be established following a rationale based on the ratio $\rho$ of enrichment concordance vs. non-concordance probabilities, $\rho = 2p_{11} / u$, with $u = p_{01} + p_{10}$. Basically, the idea is to express the Sorensen dissimilarity index (Equation 1) as a function of $\rho$:

9 $$\begin{aligned} d_S(\rho ) = \frac{1}{1 + \rho }. \end{aligned}$$

In this way, the problem reduces to stablishing a lower limit over the degree of preponderancy of coincidence over non-coincidence in enrichment. For example, taking as a reference the usual limit for the ratio of bioavailability geometric means in bioequivalence experiments [20] (addmitedly, also arbitrary and comming from a very different area of research), $\rho _0 = 10/8 = 1.25$, we have $d_0 = d_S(\rho _0) = 0.4444$, or under a less strict criterion, $d_S(10/9) = 0.4737$. [21] discusses possible equivalence limits for a ratio (and a difference and an odds-ratio) of binomial probabilities, but also in a context not directly applicable to our case. These values are based on the own definition rationale of the Sorensen–Dice index which counts twice the probability of coincidence. Alternatively, $\rho$ may be defined as $p_{11} / (p_{01} + p_{10})$. Then,

10 $$\begin{aligned} d_S(\rho ) = \frac{1}{1 + 2 \rho } \end{aligned}$$

and the ratios 10/8 and 10/9 correspond to more strict limits 0.2857 and 0.3103, respectively.

### Equivalence test for multiple comparisons

A compilation of *s* studies on a similar subject, or an experimental study on the *s* levels of a factor, may lead to a dataset formed by $L_1, ..., L_s$ gene lists. Then, it may be worth to study the equivalence of all possible pairs of lists $h = s\times (s-1)/2$, or to perform $h\le s\times (s-1)/2$ previously specified comparisons, like a “control” list vs. the remaining lists. For a given equivalence threshold $d_0$, this can be done by (i) Perform every selected comparison and (ii) Correct for testing multiplicity by means of an adequate adjusting procedure. If the number of comparisons is not big (e.g., at most some tens), one may prioritise controlling the “Family Wise Error Rate” (FWER) using for instance the Bonferroni–Holm criterion [22]:

- Compute the *p*-values $p_1(d_0), p_2(d_0), \dots , p_h(d_0)$ associated with each performed equivalence test.
- Sort the *p*-values in ascending order: $p_{(1)} \le p_{(2)} \le \dots \le p_{(h)}$,
- The null hypothesis of non-equivalence (i.e. existence of a “relevant” dissimilarity between lists) is rejected for all those comparisons $l=1, ..., k-1$ such that $p_l(d_0) < p_{(k)}(d_0)$ where *k* is the smallest value satisfying that $p_{(k)}(d_0) > \alpha / (h+1-k)$.

This is the approach that we have taken in the example case-studies of this paper.

In the case of a great number *h* of comparisons, possibly other criteria like the False Discovery Rate (FDR) [23] for multiple testing corrections would be the option to choose, but the general idea is still the same.

**Fig. 1:**
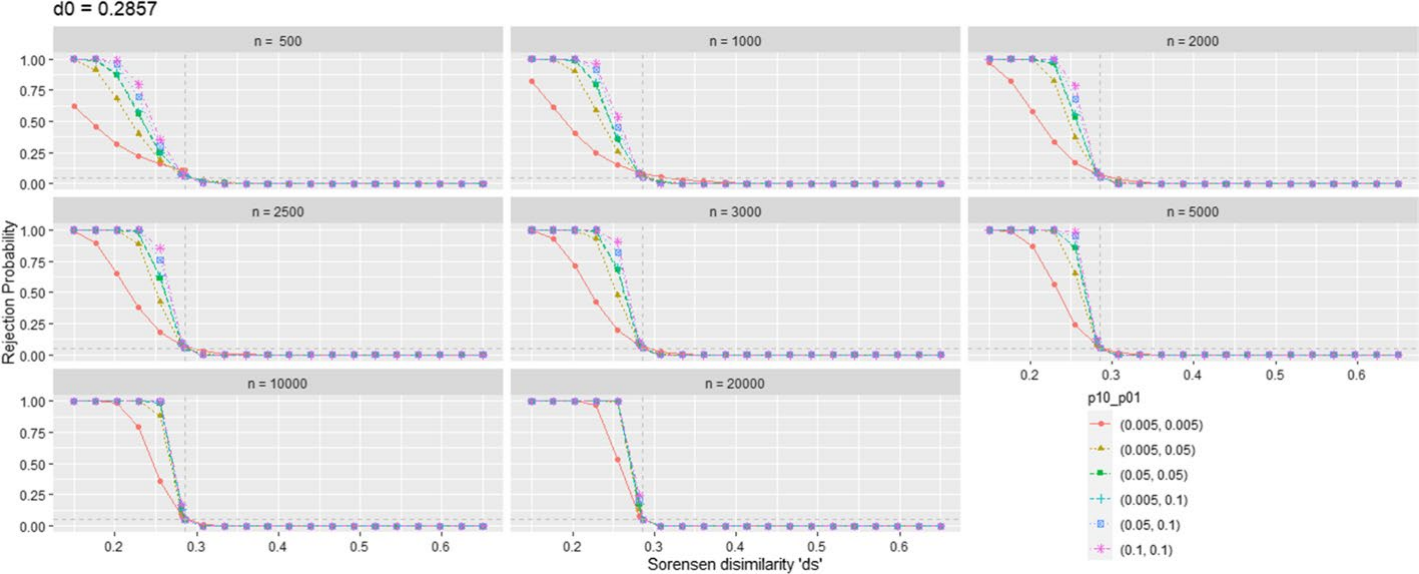
Probability of rejecting the null hypothesis of non equivalence in the Sorensen test (i.e., to declare biological similarity) with $\alpha = 0.05$

**Fig. 2:**
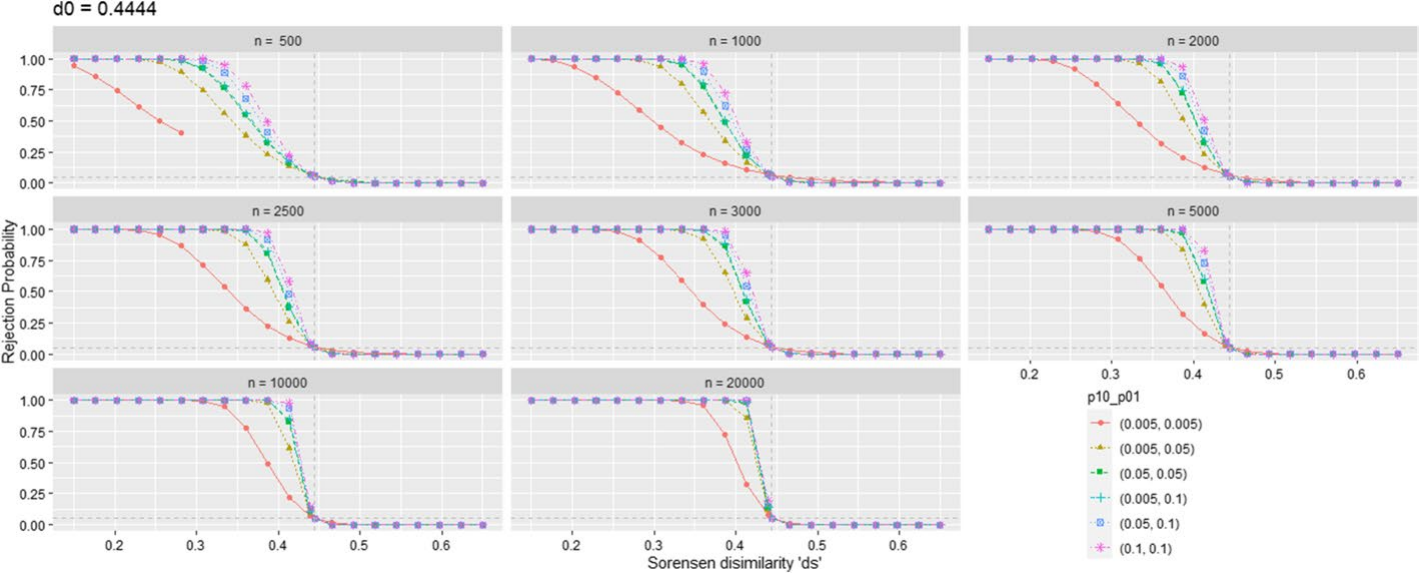
Probability of rejecting the null hypothesis of non equivalence in the Sorensen test (i.e., to declare biological similarity) with $\alpha = 0.05$

### Test validity and efficiency. Simulations and bootstrap approach

Here we describe a simulation study using the R package base [24], devoted to assess the validity of the preceding equivalence test and the asymptotic theory in which it is based. The simulation results described below are based on generating vectors $(n_{11}, n_{01}, n_{10}, n_{00})$ from a multinomial distribution of parameters *n* (total number of GO terms) and $p = (p_{11}, p_{01}, p_{10}, p_{00})$. Alternatively, with the same results, the simulated datasets may be obtained first generating the number $\nu$ of enriched terms as a binomial of parameters *n* and $Pr\{\text{E}\} = p_{11} + p_{01} + p_{10}$ and then, conditioned to the observed value of $\nu$, generating $(n_{11}, n_{10}, n_{01})$ as a multinomial of parameters $\nu$ and $\pi = (\pi _{11}, \pi _{01}, \pi _{10})$ with $\pi _{ij} = p_{ij} / Pr\{\text{E}\}$.

The simulated scenarios were the result of crossing the following levels of $d_0$, *n* and $(p_{01}, p_{10})$: $d_0 = 0.2857, 0.3103, 0.4444, 0.4737$, $n = (500, 1000, 2000, 2500, 3000, 5000,$ 10000, 20000) and $(p_{01}, p_{10}) = (0.005, 0.005), (0.005, 0.01), (0.01, 0.01), (0.005, 0.05),$ (0.01, 0.05), (0.05, 0.05), (0.005, 0.1), (0.01, 0.1),  (0.05, 0.1), (0.1, 0.1), (0.005, 0.2), (0.01, 0.2), (0.05, 0.2), (0.1, 0.2), (0.2, 0.2). Additionally, for each pair of fixed $p_{01}$ and $p_{10}$, the value of $p_{11}$ was computed as a function of a given set of desired theoretical Sorensen dissimilarities $d_S$ (some of them in the parametric region of $H_1$, i.e., $d_S < d_0$, and some of them in the parametric region of $H_0$, i.e., $d_S \ge d_0$) according to $p_{11} = (1-d_S)(p_{10} + p_{01}) / (2d_S)$, which is simply the solution of Equation 1. For each one of these scenarios, we ran $10^5$ simulation replicates.

These simulations, and the computations associated to the examples described in the next section, were carried out using the R package goSorensen which was developped by the authors in parallel to the elaboration of the paper. It is accessible at GitHub, https://github.com/pablof1988/goSorensen. From now on, for brevity, we will designate the method presented here with the same name than the package, goSorensen.

In Fig. 1 and Fig. 2, for $d_0 = 0.2857$ and $d_0 = 0.4444$ respectively, the probability (really, its precise but random simulation estimation) of rejecting $H_0$ is represented on the ordinate axis while the simulated dissimilarities $d_S$ (including the one corresponding to threshold $d_0$) are represented on the abscissa axis. Thus, the $d_S$ values on the left of $d_0$ represent scenarios with false $H_0$ (the values on the ordinate axis correspond to the power of the test), and the $d_S$ values to the right of $d_0$ represent scenarios where $H_0$ is true (Type I Error Probability).

In a distant vision of these figures, possibly we may conclude that this test behaves acceptably well. But going down to detail we detect a persistent inflation of type I error. At $d_S = d_0$, ideally, the probability of rejecting $H_0$ should be equal (or at least less) than the significance level. In fact it is persistently greater than this value. This inflation decreases with growing values of *n* and the probabilities of enrichment $Pr\{\text{E}\}$, i.e., with growing values of the expected frequency of enrichment, $E\left( \nu \right)$. For example, in the simulation scenario $d_S = d_0 = 0.2857$, $n = 1000$ and $p_{01} = p_{10} = 0.005$ ($p_{11} = 0.0125$ to have $d_S = d_0 = 0.2857$), with an expected frequency of enriched terms of 11.25, the simulation results is an unacceptable proportion of 0.0993 rejections of the null hypothesis for a nominal significance level of 0.05. For growing values of $(p_{01}, p_{10})$, this type I error probability progressively decreases, but even at $(p_{11}; p_{01}, p_{10}) = (0.5; 0.2, 0.2)$ (an abundance of enrichment quite unrealistic in practice), some slight inflation persists, with a value of 0.0538. These simulation estimations of the type I error probability were obtained with a milion of simulation replicates, in order to make them more precise. Measuring their precision with a 95% confidence interval around de estimated proportion of rejections, the error lies in the fourth decimal position, less than $\pm 0.0005$.

**Fig. 3:**
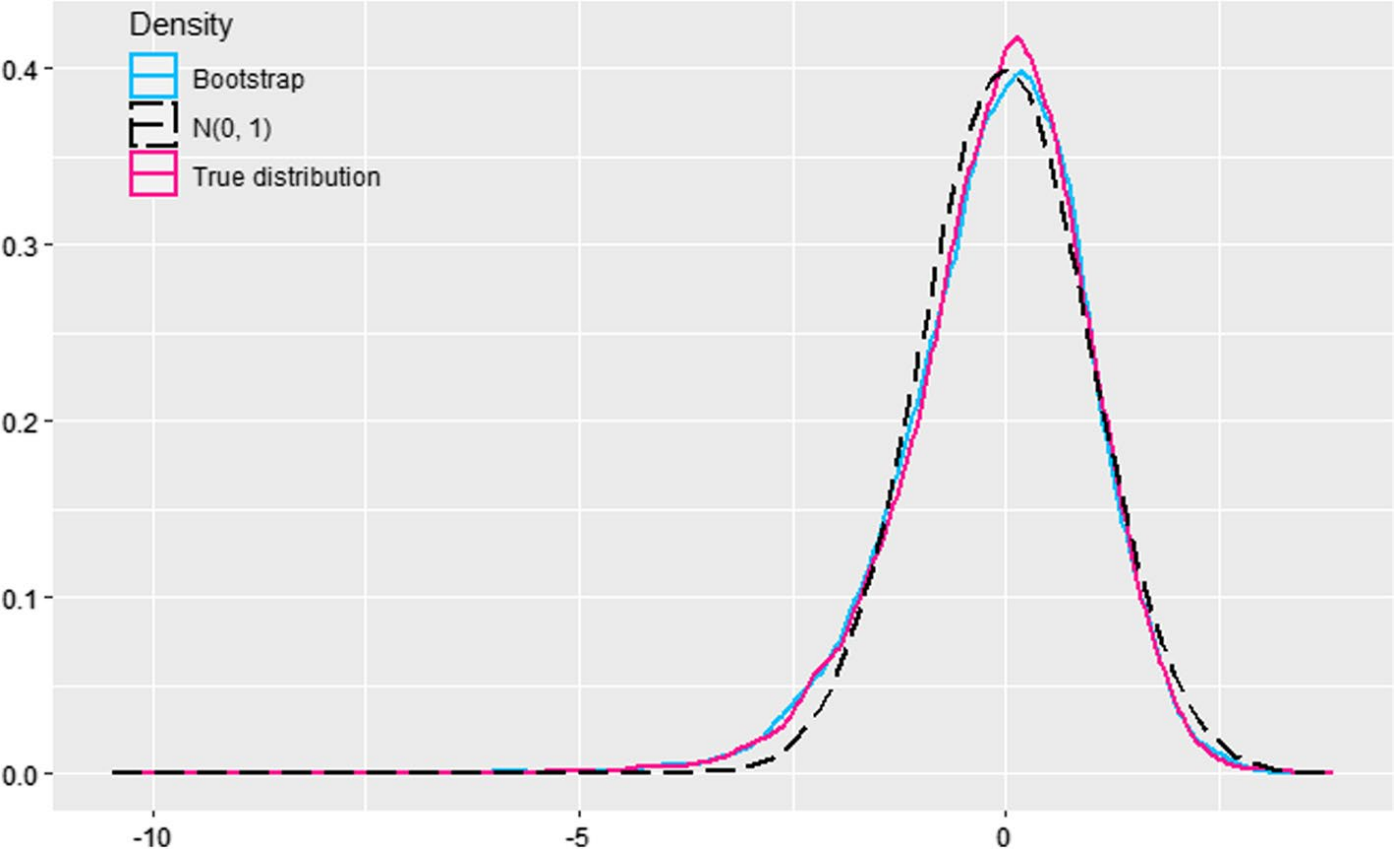
The N(0,1) density compared with the “true” distribution of the statistic ${\sqrt{n} (d_S({\hat{p}}) - d_S(p))} / {{\hat{\sigma }}_S}$ and a bootstrap estimate of its distribution

The above mentioned type I error inflation is mainly due to the slow approximation to the standard normal of the “true” sampling distribution of the studentized statistic ${\sqrt{n} (d_S({\hat{p}}) - d_S(p))} / {{\hat{\sigma }}_S}$, which has a heavier left tail than the normal. We empirically observed that the bootstrap distribution of the studentized statistic ${\sqrt{n^*} (d_S({\hat{p}}^*) - d_S({\hat{p}}))} / {{\hat{\sigma }}_S^*}$, reproduces much better this sampling distribution, left tail heaviness included. Figure 3 illustrates this fact. For $n = 1000$, $p_{01} = 0.001$, $p_{10} = 0.01$, $p_{11} = 0.01375$ and $p_{00} = 0.97525$, it displays a kernel approximation to the “true” density of the statistic ${\sqrt{n} (d_S({\hat{p}}) - d_S(p))} / {{\hat{\sigma }}_S}$ obtained from 10000 simulation replicates, the same kernel approximation from 10000 bootstrap replicates (generated from a table -chosen at random- from the 10000 tables previously simulated to obtain the “true” distribution), and the N(0,1) density.

The bootstrap distribution may be estimated from *B* simulated values of the statistic ${\sqrt{\nu ^*} (d_S({\hat{p}}^*) - d_S({\hat{p}}))} / {{\hat{\sigma }}_S^*}$ computed over *B* data tables $(n_{11}^*, n_{01}^*, n_{10}^*, n_{00}^*)$ generated from a multinomial distribution of parameters *n* and the estimated probabilities ${\hat{p}} = ({\hat{p}}_{11}, {\hat{p}}_{01}, {\hat{p}}_{10}, {\hat{p}}_{00})$, with ${\hat{p}}_{ij} = n_{ij} / n$. Equivalently, to obtain each bootstrap replicate, one may generate a value $\nu ^*$ from a binomial distribution of parameters *n* and $(n_{11} + n_{01} + n_{10}) / n$ and, conditioned to this $\nu ^*$ value, to generate $(n_{11}^*, n_{01}^*, n_{10}^*)$ from a multinomial of parameters $\nu ^*$ and $({{\hat{\pi }}}_{11}, {{\hat{\pi }}}_{01}, {{\hat{\pi }}}_{10})$, with ${{\hat{\pi }}}_{ij} = n_{ij} / \nu ^*$. An alternative procedure for bootstrapping may be to sample at random the gene lists and to construct the contingency tables from them. This is a complex and slow way so we have not considered it.

As a consequence, the confidence interval (6) may be upgraded to a bootstrap confidence interval, simply by substituting the normal quantile $z_{\alpha }$ by the empirical $\alpha$ quantile of the *B* bootstrap values ${\sqrt{\nu ^*} (d_S({\hat{p}}^*) - d_S({\hat{p}}))} / {{\hat{\sigma }}_S^*}$, and the bootstrap p-value may be computed by substituting the normal distribution function $\Phi$ by the empirical distribution of the *B* bootstrap values. The simulation results and the bootstrap computations in the examples discussed in the next section were based on $B = 10000$ bootstrap replicates.

In Tables 2 and 3 we compare the probability of type I error (the simulated dissimilarity $d_S$ is equal to $d_0$), i.e., based on the normal distribution and based on bootstrap. They are just an illustrative example corresponding to simulations with $n = 1000$ and $n = 10000$ respectively, but the general trend of the results is always the same: In the test based on the bootstrap distribution, the probability of rejecting $H_0$ is closer to the significance level $\alpha$ than in the normal test, or conservative in the problematic cases associated to low frequencies of enrichment. Similarly, in the bootstrap approach, the confidence interval coverage is closer to $1 - \alpha$. In Tables 2 and 3, the probability values corresponding to the normal test are those obtained in the simulations with $10^6$ replicates. On the other hand, the probabilities corresponding to the bootstrap approach were obtained with $10^5$ simulation runs, due to the increased slowness of this approach. But their precision is of the same order, thanks to the use of a variance reduction technique introduced at [25] and using the probabilities of the normal case as a control.

Under simulation scenarios with very low enrichment frequencies, part of the generated enrichment contingency tables are inadequate to Sorensen–Dice computations, due to the presence of zero frequencies. Then, the number of effective simulation replicates (and also the number of effective bootstrap replicates) is less than has been specified. But in these cases, while the normal test has an inflated type I error probability, the bootstrap test tends to be conservative.

**Table 2:** Probability of declaring equivalence (*pr*(*Rej*) normal test, $pr_B(Rej)$ bootstrap test) for a simulated dissimilarity equal to the equivalence limit, $d_S = d_0$. $n = 1000$ stands for the total number of GO terms, with $p_{ij}$ probabilities of enrichment.

nSim	${\mathbf{d_S}}$	${\mathbf{d_0}}$	${\mathbf{p}}_{\boldsymbol{11}}$	${\mathbf{p}}_{\boldsymbol{01}}$	${\mathbf{p}}_{\boldsymbol{10}}$	***pr***(*Rej*)	${\mathbf{pr_B(Rej)}}$	${\mathbf{E}}(\nu )$
99433	0.2857	0.2857	0.01250	0.005	0.005	0.0807	0.0251	22.50
99994	0.2857	0.2857	0.01875	0.005	0.010	0.0741	0.0371	33.75
100000	0.2857	0.2857	0.02500	0.010	0.010	0.0706	0.0417	45.00
100000	0.2857	0.2857	0.06875	0.005	0.050	0.0617	0.0485	123.75
100000	0.2857	0.2857	0.07500	0.010	0.050	0.0614	0.0483	135.00
100000	0.2857	0.2857	0.12500	0.050	0.050	0.0591	0.0500	225.00
100000	0.2857	0.2857	0.13125	0.005	0.100	0.0584	0.0493	236.25
100000	0.2857	0.2857	0.13750	0.010	0.100	0.0583	0.0496	247.50
100000	0.2857	0.2857	0.18750	0.050	0.100	0.0568	0.0499	337.50
100000	0.2857	0.2857	0.25000	0.100	0.100	0.0558	0.0497	450.00
100000	0.2857	0.2857	0.25625	0.005	0.200	0.0558	0.0498	461.25
100000	0.2857	0.2857	0.26250	0.010	0.200	0.0559	0.0497	472.50
100000	0.2857	0.2857	0.31250	0.050	0.200	0.0548	0.0494	562.50
100000	0.2857	0.2857	0.37500	0.100	0.200	0.0541	0.0490	675.00
100000	0.2857	0.2857	0.50000	0.200	0.200	0.0538	0.0495	900.00

$E(\nu )$ is the expected total number of enriched terms. *nsim* corresponds to the number of effective simulation replicates (over an initial number of $10^5$) to obtain $pr_B(Rej)$ ($nsim \times B$ test computations, $B = 10000$; *pr*(*Rej*) was based on an initial number of $10^6$ simulation replicates). In some scenarios with low $p_{ij}$, the generated tables contained zeros making impossible the Sorensen–Dice computations, so the effective number of simulation replicates was lower than what was initially planned

**Table 3:** Probability of declaring equivalence (*pr*(*Rej*) for the normal test, $pr_B(Rej)$ for the bootstrap test) when the simulated dissimilarity is equal to the equivalence limit, $d_S = d_0$.

nSim	$d_S$	$d_0$	$p_{11}$	$p_{01}$	$p_{10}$	*pr*(*Rej*)	$pr_B(Rej)$	$E(\nu )$
100000	0.2857	0.2857	0.01250	0.005	0.005	0.0590	0.0498	225.00
100000	0.2857	0.2857	0.01875	0.005	0.010	0.0570	0.0499	337.50
100000	0.2857	0.2857	0.02500	0.010	0.010	0.0560	0.0502	450.00
100000	0.2857	0.2857	0.06875	0.005	0.050	0.0534	0.0485	1237.50
100000	0.2857	0.2857	0.07500	0.010	0.050	0.0532	0.0500	1350.00
100000	0.2857	0.2857	0.12500	0.050	0.050	0.0527	0.0503	2250.00
100000	0.2857	0.2857	0.13125	0.005	0.100	0.0524	0.0499	2362.50
100000	0.2857	0.2857	0.13750	0.010	0.100	0.0524	0.0502	2475.00
100000	0.2857	0.2857	0.18750	0.050	0.100	0.0522	0.0503	3375.00
100000	0.2857	0.2857	0.25000	0.100	0.100	0.0519	0.0502	4500.00
100000	0.2857	0.2857	0.25625	0.005	0.200	0.0518	0.0501	461.25
100000	0.2857	0.2857	0.26250	0.010	0.200	0.0516	0.0501	4725.00
100000	0.2857	0.2857	0.31250	0.050	0.200	0.0513	0.0499	5625.00
100000	0.2857	0.2857	0.37500	0.100	0.200	0.0513	0.0499	6750.00
100000	0.2857	0.2857	0.50000	0.200	0.200	0.0512	0.0501	9000.00

$n = 10000$ stands for the total number of GO terms, with $p_{ij}$ probabilities of enrichment. $E(\nu )$ is the expected total number of enriched terms. *nsim* corresponds to the number of effective simulation replicates to obtain $pr_B(Rej)$ ($nsim \times B$ test computations, $B = 10000$; *pr*(*Rej*) was based on $10^6$ simulation replicates)

## Results

### Cancer gene lists, allOnco

Our fist example is based on the gene lists compiled at http://www.bushmanlab.org/links/genelists, a comprehensive set of gene lists related to cancer (allOnco). The exact lists, and the genes constituting each list, were the same analysed at [12] with the goProfiles method. These lists were Atlas, Cangenes, Cis, Miscellaneous, Sanger, Vogelstein and Waldman.

They were selected with the criterion of discarding small lists (less than 100 genes). This requirement for “large” gene lists is related to assure the validity of the goProfiles method, further considered for the sake of the comparison with the method presented here, goSorensen. In fact, the sample size of goSorensen is finally given by the number of GO terms under consideration and the number of enriched GO terms.

Given its origin, there were’nt any a priori expectations on which lists should be mutually equivalent. Provided its heterogeneous origin, not many equivalencies were expected at low equivalence limits. Here we describe the results of the equivalence analyses performed under $d_0 = 0.4444$ and $d_0 = 0.2857$. As assessed in Methods section, both equivalence limits correspond to the same degree of preponderancy of joint enrichment $p_{11}$ over $p_{10} + p_{01}$, the probability of one GO term being enriched in one list but not in the other. In $d_0 = 0.4444$, $p_{11}$ is counted twice (possibly on line with the definition criteria of the Sorensen–Dice index) while in $d_0 = 0.2857$ not.

We performed equivalence analyses separately for each GO ontology and for GO levels 3–10. For each pair of gene lists, the contingency table of joint enrichment (see Table 1) was built under a cutoff p-value of 0.01 and a cutoff q-value of 0.05. The equivalence tests were performed under a significance level $\alpha = 0.05$ and correcting for testing multiplicity by means of the Holm’s method, as is suggested in Equivalence test for multiple comparisons section. The equivalence analyses were performed first using the normal asymptotic variant of the test and subsequently with the bootstrap variant, with coincidental results: With few exceptions, the end conclusion of the normal and the bootstrap test was the same, with greater *p*-values in the bootstrap case, as is expected. All tests were performed under Bioconductor version 3.13 and R versions 4.1.0 and 4.1.1. The results may present some minor differences under other Bioconductor versions.

These analyses provide considerably stable results along nearly all GO levels, and interesting regularities across the three ontologies. Here we focus on the statistical analyses of these data, as an illustrative operational case-study and not particularly in their biological interpretation. The R code performing all the analyses can be accessed at sorensenEquivScripts GitHub repository, available in https://github.com/pablof1988/sorensenEquivScripts.

For the BP ontology, with the equivalence limit $d_0 = 0.4444$, the gene lists Atlas, Miscellaneous, Sanger, Vogelstein and Waldman constitute a stable group of equivalent gene lists. For a more restrictive equivalence limit $d_0 = 0.2857$, declaring equivalence between lists is a much more rare event: It is declared only between Sanger and Vogelstein, along all GO levels.

**Fig. 4:**
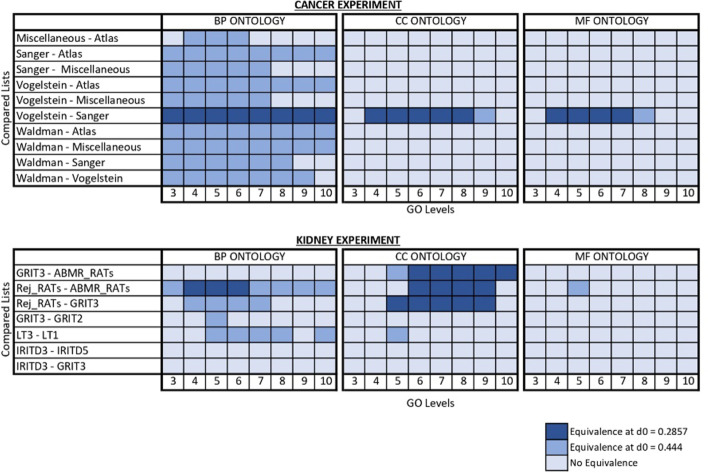
Equivalences between gene lists

The CC and MF ontologies are less adequate for the study of these gene lists with the present method, the incidence of enrichment is lower than in the BP ontology and thus the validity of the asymptotic results may be doubtful at some GO levels. But in general the bootstrap test, more conservative, corroborates the same ubiquitous equivalencies between Sanger and Vogelstein. Figure 4 tries to graphically summarize all these equivalencies. The complete listing of detected equivalences can be found in the Additional file 2.

Provided that the methods commonly used in the analysis of gene (or in general, feature) lists are essentially descriptive, it is hard to compare the inferential method presented here with them. The most obvious match is the before cited goProfiles method. It is based on the squared Euclidean distance, $d^2_E$, a dissimilarity which takes values in a much more variable scale than the Sorensen–Dice dissimilarity $d_S$. Thus, for comparative purposes it is difficult to numerically establish equivalence limits comparable to those used above. On the other hand, given a set of gene lists, [12] introduced an iterative method, and its associated graphical representation, to obtain the full scale of equivalence limits ranging from zero to the smallest limit that would make all lists equivalent. Let us designate it as $d^2_{Emax}$. Larger equivalence limits do not make a great deal of sense in the goProfiles method. Obviously, in the range from zero to $d^2_{Emax}$ it is included the smallest equivalence limit that would make equivalent only the two nearest gene lists and the remaining admissible equivalence limits. Just for operative purposes, we will discuss the detected equivalencies between lists for two equivalence limits: 10% and 20% of $d^2_{Emax}$.

The preceding results for the goProfiles method are considerably consistent with those in the supplementary material of [12] for the goProfiles method. These goProfiles results, updated to Bioconductor 3.13, are available at https://github.com/pablof1988/goProfilesSupplementary. Here we outline the main results:

For the most restrictive equivalence limit (i.e., $0.1 d^2_{Emax}$), the equivalency between Sanger and Vogelstein also emerges as ubiquitous along all GO ontologies and levels. In the BP ontology, under the less restrictive equivalence limit $0.2 d^2_{Emax}$), the equivalencies between all lists in the group Atlas, Sanger, Vogelstein and Waldman constitute a commonly repeated pattern. Also in the BP ontology, Cangenes and Cis are declared equivalent for GO levels 3 to 8 and Atlas and Cis at all GO levels The equivalency between Miscellaneous and Waldman is also common to all GO levels (sometimes even for the most restrictive limit), and not ubiquitous but frequent at many levels in the CC and MF ontologies.

In the supplementary material of [12], these data were analysed also by means of the Semantic Similarity method, e.g., [26], using the package GOSemSim [27, 28]. In all the variants of this method that were considered, Sanger and Vogelstein were the closest lists, followed by Miscellaneous and Waldman.

So, there is a low expectancy of test results providing evidence of equivalency (for those methods based on an inferential equivalence testing approach) or descriptive similarities between the allOnco gene lists, but the clear ones are considerably consistent along analytical approaches.

### Pathogenesis-based transcripts sets (PBTs)

Our second case study is based on the pathogenesis-based transcripts sets (PBTs) available at https://www.ualberta.ca/medicine/institutes-centres-groups/atagc/research/gene-lists.html. Citing textually this source, these gene lists were collected “to represent the major biologic events in cellular graft rejection, cytotoxic T-cell infiltration, interferon-gamma effects and epithelial deterioration”. More especifically, our dataset is a subset of the so-called “Core PBT List (HG U219 arrays)” containing gene lists related to kidney rejection after transplantation events. We analysed a subset of 14 gene lists. Using the list names in the before cited web, we analysed the lists ABMR-RATs, BAT, CT1, ENDAT, GRIT2, GRIT3, IRITD1, IRITD3, IRITD5, KT1, LT1, LT3, Rej-RATs and TCMR-RATs. Readers can find additional detail on their biological meaning in the web. As in the previous example, these lists were selected with the criterion of discarding small lists (less than 100 genes). Not all these gene lists are directly related to kidney transplantation but their inclusion makes sense in a comparative study. For example, LT1 and LT2 are lung-specific analogous to the kidney transcript sets KT1 and KT2 (this last one not included in the analyses). All equivalence tests were performed under the same settings than for the allOnco cancer gene lists. The complete listing of detected equivalencies can be found in the Additional file 2.

In the BP ontology, for the $d_0 = 0.4444$, lists ABMR-RATs and Rej-RATs were declared equivalent, consistenly along GO levels 3 to 10 and by both variants of the test. The same result is also applicable to Rej-RATs and GRIT3, but only along levels 4 to 7. There are some occasional equivalencies of dubious interest. For the more restrictive value $d_0 = 0.2857$, equivalence between ABMR-RATs and Rej-RATs was the only one declared, only for GO levels 4, 5 and 6.

In the CC and MF ontologies, the frequencies of enrichment were very low. Then, the conservative bootstrap approach seems the only reliable option. In the CC ontology, for both equivalence limits under consideration, $d_0 = 0.2857, 0.4444$ equivalencies were declared consistently inside the group ABMR-RATs, Rej-RATs and GRIT3, for the GO levels 6 to 9. In the MF ontology, the equivalency between ABMR-RATs and Rej-RATs is the only one detected, at level 5 and for $d_0 = 0.4444$.

When the same data are analysed by means of the goProfiles approach, with the same equivalence limits as before (i.e., the less stringent $0.2 d^2_{Emax}$ and the tighter $0.1 d^2_{Emax}$), there appears a rich structure of equivalencies. The R code to obtain them can be accessed at goProfilesSupplementary GitHub repository, available in https://github.com/pablof1988/goProfilesSupplementary.

Here we outline the main equivalencies detected by the goProfiles method: The equivalence between LT1 and LT2 is ubiquitous along all ontologies and levels, most frequently at the more restrictive equivalence limit. As mentioned, these lists refer to lung rejection events, included for the sake of the comparison. More interestingly, there are equivalencies between these two lists and other kidney rejection lists: In the BP ontology, the lists CT1 and KT1 are also mutually equivalent and constitute a group of four equivalent lists with LT1 and LT2, at all GO levels (for levels 3 to 8 at the most restrictive limit). In the MF ontology, the same group of four equivalent lists is detected (GO levels 3, 4, 6, 7, 8, 9) with diverse additional equivalent members although without a clear pattern. Also in all the ontologies, there is equivalency between IRITD3 and IRITD5, most frequently at the most restrictive equivalence limit. In the CC ontology, lists ABMR-RATS and Rej-RATS are consistently equivalent along many GO levels, chiefly at the most restrictive equivalence limit.

**Fig. 5:**
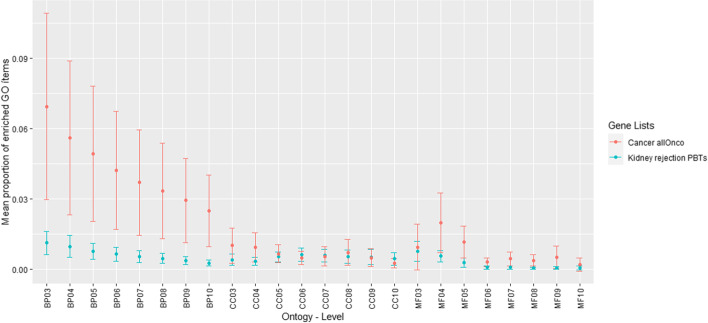
Average proportion of enriched GO terms in the Kidney rejection PBTs and Cancer allOnco gene lists, displayed along GO ontologies and GO levels

## Discussion

We would like to start by stating an apparently negative result: For the kidney rejection PBTs data, the performance of goSorensen is somewhat deceiving, especially when it is compared with the performance of the goProfiles approach for the same dataset. Figure 5 illustrates why in the PBTs data goSorensen performs worse than goProfiles. It also provides a first hint on when it might be worth using goSorensen: Use it only if the lists are well annotated and GO terms enrichment is relevant to characterize the genes in a list. Figure 5 displays the average proportion of enriched terms along all gene lists of the PBTs dataset and compares it with the same summary for the gene lists in the allOnco dataset. These averages are displayed along all GO ontologies and levels under consideration. It seems clear that GO terms enrichment incidence is extremely low in the PBTs dataset.

To get a first global impression on the relation with both approaches (goProfiles and goSorensen), one may put in relation the outcome of the equivalence test described in Equivalence test subsection with the equivalence test defined in expressions (5) and (6) of [12]. Given ${L_1}, \ldots ,{L_s}$ gene lists, the output of the corresponding tests can be represented as triangular matrices like

$$\begin{aligned}X = \left( {\begin{array}{*{20}{c}} {{x_{12}}}&{}{}&{}{}\\ \vdots &{} \ddots &{}{}\\ {{x_{1s}}}&{} \ldots &{}{{x_{s - 1,s}}} \end{array}} \right) {\mathrm{and}}\, Y = \mathrm{{ }}\left( {\begin{array}{*{20}{c}} {{y_{12}}}&{}{}&{}{}\\ \vdots &{} \ddots &{}{}\\ {{y_{1s}}}&{} \ldots &{}{{y_{s - 1,s}}} \end{array}} \right) \end{aligned}$$

where ${x_{ij}}$ and ${y_{ij}}$ stand for an adequate test output, with ${x_{ij}}$ referring to the test presented here and ${y_{ij}}$ to the goProfiles test. Examples of adequate candidates for ${x_{ij}}$ and ${y_{ij}}$ are: (i) The upper limit of the one-sided confidence interval like 6, (ii) The test p-value, and (iii) A label for the test outcome as “not reject the null hypothesis” and “reject the null hypothesis” (or 0 and 1). The last two quantities depend on the chosen equivalence limits which makes comparison difficult as both distances are on very different scales. We use the confidence interval upper limit (asymptotic normal test for the method introduced here, the results are very similar for the bootstrap test) as a more objective quantity: In their respective scales, in both tests large values will tend to correspond to not declaring equivalence and small values to declaring equivalence. Mantel’s test, [29], is adequate to study the significance of the correlation between triangular matrices like *X* and *Y*.

Table 4 empirically suggests that there is a considerable positive estimated correlation (values between 0.6 and 0.8, with the exception of level 3 in ontology MF) between the goProfiles/Squared-Euclidean-Distance and the goSorensen/Enrichment-Tables/Sorensen-Dissimilarity equivalence tests when both are applied to the allOnco cancer lists dataset. There appears to be a general tendency of one of them to declare equivalence as the other also declares it, and conversely. On the other hand, for the kidney rejection PBTs gene lists, the correlations between the outcome of both testing approaches are much lower, ranging from approximately 0.1 to 0.4. For both datasets, according to the Mantel’s test, these correlations tend to be statistically significant i.e., the null hypothesis of null correlation would be rejected at a “standard” (but arbitrary) level like 0.05. This a clear example of the difference between “statistically significant” and “important”: For the PBTs data, even a very low (but possibly non-null) correlation gives a significant result for a large sample size of $14(14 - 1) / 2 = 91$ values. There is some positive correlation but it is very low.

Table 5 helps to understand the basis of this relationship, or its absence. In the goProfiles approach, the decision is based on the annotation frequencies of the GO terms under consideration and in a dissimilarity index based on the mutual differences between these frequencies. In Table 5 the GO terms under consideration are arranged in the following way: First, all those non-enriched in both lists, next those enriched in the first list but not in the second, next those enriched in the second one but not in the first and finally those enriched in both lists. The order in which the GO terms are displayed is not relevant in goProfiles but clarifies the relationship of the goProfiles approach with what is relevant in the method now under consideration. In it, the frequencies are substituted by zero and one values, in correspondence with the non-enriched/enriched status of these GO terms and the sum of these zeros and ones conducts to the enrichment contingency table. For high levels of incidence of the GO terms enriched status (high ${\hat{p}}_{11}, {\hat{p}}_{01}$ and ${\hat{p}}_{10}$):

- Terms non enriched in both lists are not considered by the Sorensen–Dice dissimilarity and presumably they have also a low contribution to squared Euclidean distances, due to low frequencies of annotation (in general, enrichment is associated to high frequencies of annotation).
- Terms enriched in one list but not in the other tend contribute to higher dissimilarities for both indexes: For the squared Euclidean distance they tend to correspond to high frequencies in one list and low frequencies in the other and so to high differences. For the Sorensen–Dice index, abundance of these terms would contribute to high values of ${\hat{p}}_{01}$ and ${\hat{p}}_{10}$, and so to high dissimilarities.
- Terms enriched in both lists contribute to lower values for the Sorensen dissimilarity although the relation with the squared Euclidean distance is not so clear, one term may be enriched in both lists but with different (in general large) frequencies.

Then, when abundance of enriched terms is important on characterizing some gene lists, we can also expect some degree of coincidence between the dissimilarity indexes in which both methods are based. If not, the annotation frequencies may display also some patterns which are captured by the goProfiles method but not by the enrichment-based method. The allOnco cancer gene lists seem to correspond to the first scenario while the PBTs kidney rejection lists seem to correspond to the second scenario. This is corroborated by Fig. 5 which displays high incidences of enrichment for the cancer lists and very low incidence for the kidney rejection lists.

**Table 4:** Degree of coincidence between the equivalence test described here and the equivalence test based on the goProfiles approach.

Onto Level	AllOnco gene lists		PBT’s gene lists	
	Correlation	*p* - value	Correlation	*p* - value
BP-3	0.6507	0.0022	0.2711	0.0013
BP-4	0.6895	0.0008	0.3781	0
BP-5	0.6943	0.0004	0.372	0
BP-6	0.6703	0.0006	0.3214	0.0004
BP-7	0.66	0.0004	0.2777	0.0019
BP-8	0.6409	0.0004	0.238	0.0082
BP-9	0.704	0.0002	0.2026	0.0229
BP-10	0.7178	0.0002	0.196	0.0282
CC-3	0.5199	0.0036	0.151	0.0683
CC-4	0.54	0.0022	0.1753	0.0386
CC-5	0.5648	0.001	0.3057	0
CC-6	0.4052	0.006	0.2354	0.0017
CC-7	0.3964	0.0089	0.2127	0.0071
CC-8	0.4671	0.0073	0.1795	0.0318
CC-9	0.5888	0.0085	0.2083	0.0046
CC-10	0.7008	0.0032	0.2878	0
MF-3	0.3878	0.0556	0.1088	0.1825
MF-4	0.6514	0.0018	0.1303	0.1051
MF-5	0.6437	0.002	0.1906	0.0208
MF-6	0.7292	0.0002	0.1929	0.0103
MF-7	0.7539	0.0002	0.0735	0.3016
MF-8	0.601	0.0018	0.2117	0.0193
MF-9	0.4453	0.0167	0.1629	0.0244
MF-10	0.1874	0.0667	0.4846	0.0476

The correlations were computed over the upper limits of the one-sided confidence intervals defining the tests. These upper limit values were organized as triangular matrices (upper limit $x_{ij}$ when testing list *i* vs. list *j* with one test, $y_{ij}$ for the other test) for the kidney transplantation rejection and cancer datasets. Its significance was stablished by means of the Mantel’s test

**Table 5:** Comparing the data structures to compute the goProfiles test and the one based on enrichment contingency tables.

	Non-enriched in both lists			Enriched only in list 1			Enriched only in list 2			Enriched in both lists		
GO term number	1	$\cdots$	$a = n_{00}$	$a + 1$	$\cdots$	$b = a + n_{10}$ $= n_{.0}$	$b + 1$	$\cdots$	$c = b + n_{01}$	$c + 1$	$\cdots$	$c + n_{11}$ $= n$
Annotation frequency in gene list 1	$F_{11}$	$\cdots$	$F_{1a}$	$F_{1(a + 1)}$	$\cdots$	$F_{1b}$	$F_{1(b+1)}$	$\cdots$	$F_{1c}$	$F_{1(c+1)}$	$\cdots$	$F_{1n}$
Annotation frequency in gene list 2	$F_{21}$	$\cdots$	$F_{2a}$	$F_{2(a + 1)}$	$\cdots$	$F_{2b}$	$F_{2(b+1)}$	$\cdots$	$F_{2c}$	$F_{2(c+1)}$	$\cdots$	$F_{2n}$
Enrichment in list 1	0	$\cdots$	0	1	$\cdots$	1	0	$\cdots$	0	1	$\cdots$	1
Enrichment in list 2	0	$\cdots$	0	0	$\cdots$	0	1	$\cdots$	1	1	$\cdots$	1

In the latter test, the annotation frequencies are substituted by 0 and 1 (i.e., “non-enriched” and “enriched” GO term.) and if the test is based on the Sorensen–Dice similarity, the first set of GO terms (non-enriched in both lists) is ignored. The GO terms are arbitrarily ordered: from left to right, first there are all those non-enriched in both lists ($n_{00}$ in total), next those enriched in the first list but not in the second one ($n_{10}$), then those enriched in the second list but not in the first ($n_{01}$) and finally those GO terms enriched in both lists ($n_{11}$)

## Conclusions

Summing up, both methods, goProfiles and goSorensen, reflect interesting characteristics of gene lists which may provide valuable biological information, with their pros and cons.

Aside having an inferential basis, i.e., providing some hints on the “statistical significance” of the results, in our opinion the main strength of the present method is that it is based on a concept, enrichment, which is widely used and understood. Its main weakness, is that establishing the enrichment status of a GO term adds an extra amount of uncertainty to the analysis: It depends on the output of an hypotheses test, which in turn depends on previous decisions like the significance levels under consideration to decide enrichment or the method to cope with testing multiplicity. On the other hand, the goProfiles approach is based on more objective data: the raw frequencies of annotation, which have proved to be useful but perhaps harder to interpret. This increased difficulty of interpretation is also associated to the most variable scale of values of the squared Euclidean distance over the Sorensen–Dice dissimilarity, which makes harder to stablish dependable equivalence limits.

With respect to its scope of application, goSorensen is adequate for gene lists with high levels of annotation, provided that both inferential approaches supporting it, delta method and bootstrap, are intrinsically asymptotic. In other words, the method is adequate when the projection of the gene lists in the GO is translated into a great number of GO terms, particularly in the case of its asymptotic normal version, which is associated to some danger of detecting false equivalencies. On the other hand, in these scenarios (low annotation) the bootstrap version tends to be conservative, with some risk of not detecting truly equivalent lists.

## Supplementary Information

**Additional file 1:** Appendix

**Additional file 2:** goSorensen results for allOnco and PBTs lists.

## Data Availability

goSorensen package is available at github https://github.com/pablof1988/goSorensen Pathogenesis-based transcripts sets (PBTs) lists are available at https://www.ualberta.ca/medicine/institutes-centres-groups/atagc/research/gene-lists Cancer Gene lists are available at http://www.bushmanlab.org/links/genelists R code to analyse Cancer and PBT’s lists are available at https://github.com/pablof1988/sorensenEquivScripts Results using goProfiles methods are available at https://github.com/pablof1988/goProfilesSupplementary.

## Abbreviations

BP: Biological process
CC: Cellular component
FDR: False discovery rate
FWER: Family-wise error rate
GO: Gene ontology
GOID: GO term identifier
GSEA: Gene set enrichment analysis
MF: Molecular function
PBTs: Pathogenesis-based transcripts sets.
