# Supplementary material for: An equivalence test between features lists, based on the Sorensen–Dice index and the joint frequencies of GO term enrichment
Source: BMC Bioinformatics. 2022 May 31;23:207. doi: 10.1186/s12859-022-04739-2 (PMC9158181; doi:10.1186/s12859-022-04739-2)
Supplement: Supplementary file 1 — Additional file 1: Appendix [file 12859_2022_4739_MOESM1_ESM.pdf]

# Appendix

## Software

The simulations and the application case-studies presented in this paper were performed using the R package goSorensen which was developed by two of the authors (Flores and Ocaña) during the elaboration of the paper. It is accessible at GitHub, https://github.com/pablof1988/goSorensen.
For the moment, the goSorensen package provides the following functions:

- **buildEnrichTable** Build an enrichment contingency table from two gene lists.
- **nice2x2Table** Check for validity an enrichment contingency table.
- **dSorensen** Compute the Sorensen-Dice dissimilarity.
- **seSorensen** Standard error estimate of the sample Sorensen-Dice dissimilarity.
- **duppSorensen** Upper limit of a one-sided confidence interval (0,dUpp] for the population dissimilarity.
- **equivTestSorensen** Equivalence test between two gene lists, based on the Sorensen-Dice dissimilarity.
- **allEquivTestSorensen** Iterate equivTestSorensen along GO ontologies and GO levels.
- **getDissimilarity, getPvalue, getSE, getTable, getUpper, getNboot** Accessor functions to some fields of an equivalence test result
- **upgrade** Updating the result of an equivalence test, e.g., changing the equivalence limit.

All these functions are generic, with methods for classes representing diverse kinds of data or statistical results. dSorensen, seSorensen, duppSorensen and equivTestSorensen have methods to manage classes directly representing enrichment cross-tabulations or, alternatively, character vectors representing gene lists. In this second case (gene lists), the enrichment tables are built in a previous step. If the first parameter of these functions is a list of character vectors (i.e., a list of gene lists) all paired enrichment cross-tabulations are performded and the result is a symmetric matrix of all paired dissimilarities, or standard

errors, or confidence interval upper limits -or a list structure emulating a symmetric matrix as a result of equivTestSorensen. The accessor and upgradding functions have methods for all classes of equivTestSorensen and allEquivTestSorensen results.

The R code to obtain the results of the case-studies discussed in the paper is based on package goSorensen and is available at https://github.com/pablof1988/sorensenEquivScripts.

## Mathematical details

Table 1: Contingency table for frequencies of enriched and non enriched GO terms in two gene lists $L_1$ and $L_2$

| | **Enriched in $L_2$** | **Non enriched in $L_2$** | |
|---|---|---|---|
| **Enriched in $L_1$** | $n_{11}$ | $n_{10}$ | $n_{1.}$ |
| **Non enriched in $L_1$** | $n_{01}$ | $n_{00}$ | $n_{0.}$ |
| | $n_{.1}$ | $n_{.0}$ | $n$ |

As a starting point, we assume that the underlying probabilistic model associated to frequencies $(n_{11}, n_{01}, n_{10}, n_{00})$ like those represented in Table **??** is a multinomial distribution:

$$p(n_{11}, n_{01}, n_{10}, n_{00}) = \frac{n!}{n_{11}!\ n_{01}!\ n_{10}!\ n_{00}!} p_{11}^{n_{11}} p_{01}^{n_{01}} p_{10}^{n_{10}} p_{00}^{n_{00}}$$

and so $\sqrt{n}\,(\hat{p} - p)$ is asymtotically normal $N\,(0, \Sigma)$, with

$$\Sigma = \begin{pmatrix} p_{11}\,(1 - p_{11}) & -p_{11}p_{01} & -p_{11}p_{10} \\ -p_{11}p_{01} & p_{01}\,(1 - p_{01}) & -p_{01}p_{10} \\ -p_{11}p_{10} & -p_{01}p_{10} & p_{10}\,(1 - p_{10}) \end{pmatrix}.$$

Then, as a direct consequence of the delta method,

$$\sqrt{n}\,(d_S\,(\hat{p}) - d_S\,(p)) \simeq (t_{11}, t_{01}, t_{10}) \begin{pmatrix} \hat{p}_{11} - p_{11} \\ \hat{p}_{01} - p_{01} \\ \hat{p}_{10} - p_{10} \end{pmatrix},$$

where the $t_{ij}$ stand for the partial derivatives of $d_S\,(\hat{p})$ at $p$:

$$\begin{array}{rcl} t_{11} = \left.\frac{\partial d_S(\hat{p}_{11})}{\partial \hat{p}_{11}}\right|_{p_{11}} & = & \frac{2(p_{01}+p_{10})}{(2p_{11}+p_{01}+p_{10})^2} \\ t_{01} = \left.\frac{\partial d_S(\hat{p}_{01})}{\partial \hat{p}_{01}}\right|_{p_{01}} & = & \frac{-2p_{11}}{(2p_{11}+p_{01}+p_{10})^2} = t_{10} \end{array}.$$

Then, $\sqrt{n}\,(d_S\,(\hat{p}) - d_S\,(p))$ is asymptotically normal with variance

$$\sigma_S^2 = (t_{11}, t_{01}, t_{10}) \begin{pmatrix} p_{11}\,(1 - p_{11}) & -p_{11}p_{01} & -p_{11}p_{10} \\ -p_{11}p_{01} & p_{01}\,(1 - p_{01}) & -p_{01}p_{10} \\ -p_{11}p_{10} & -p_{01}p_{10} & p_{10}\,(1 - p_{10}) \end{pmatrix} \begin{pmatrix} t_{11} \\ t_{01} \\ t_{10} \end{pmatrix}$$

$$= (t_{11}, t_{01}, t_{10}) \begin{pmatrix} p_{11} & 0 & 0 \\ 0 & p_{01} & 0 \\ 0 & 0 & p_{10} \end{pmatrix} \begin{pmatrix} t_{11} \\ t_{01} \\ t_{10} \end{pmatrix}$$

$$+\ (t_{11}, t_{01}, t_{10}) \begin{pmatrix} -p_{11}^2 & -p_{11}p_{01} & -p_{11}p_{10} \\ -p_{11}p_{01} & -p_{01}^2 & -p_{01}p_{10} \\ -p_{11}p_{10} & -p_{01}p_{10} & -p_{10}^2 \end{pmatrix} \begin{pmatrix} t_{11} \\ t_{01} \\ t_{10} \end{pmatrix}.$$

These matrix operations conduct to

$$\sigma_S^2 = t_{11}^2 p_{11} + t_{01}^2 p_{01} + t_{10}^2 p_{10} + \left( \underbrace{t_{11}p_{11} + t_{01}p_{01} + t_{10}p_{10}}_{=0} \right)^2 .$$

and finally:

$$\begin{aligned} \sigma_S^2 &= \frac{4(p_{01}+p_{10})^2}{(2p_{11}+p_{01}+p_{10})^4} p_{11} + \frac{4p_{11}^2}{(2p_{11}+p_{01}+p_{10})^4} (p_{01}+p_{10}) \\ &= \frac{4p_{11}(p_{01}+p_{10})(p_{11}+p_{01}+p_{10})}{(2p_{11}+p_{01}+p_{10})^4}. \end{aligned}$$

This variance can be estimated as:

$$\widehat{\sigma_S^2} = \frac{4\hat{p}_{11}(\hat{p}_{01}+\hat{p}_{10})(\hat{p}_{11}+\hat{p}_{01}+\hat{p}_{10})}{(2\hat{p}_{11}+\hat{p}_{01}+\hat{p}_{10})^4}.$$

As has been mentioned in **Test validity and efficiency** subsection, an equivalent alternative probabilistic model consists in assuming that the random number $\nu$ of enriched terms follows a binomial of parameters $n$ and $Pr\{\mathrm{E}\} = p_{11}+p_{01}+p_{10}$ and then, conditioned to having observed $\nu$, $(n_{11}, n_{10}, n_{01})$ follows a multinomial of parameters $\nu$ and $\pi = (\pi_{11}, \pi_{01}, \pi_{10})$ with $\pi_{ij} = p_{ij}/Pr\{\mathrm{E}\}$.

The Sorensen-Dice index may be stated, indiferently, in terms of the unconditional probabilities, $p_{ij}$, or the conditional probabilities, $\pi_{ij}$ as they differ only by a multiplicative constant, $p_{ij} = \pi_{ij}/Pr\{\mathrm{E}\}$. As a consequence, it makes no difference if one expresses this dissimilarity in terms of these conditional probabilities or in terms of unconditional probabilities:

$$d_S(\pi) = 1 - \frac{2\pi_{11}}{2\pi_{11}+\pi_{10}+\pi_{01}} = 1 - \frac{2p_{11}}{2p_{11}+p_{10}+p_{01}} = d_S(p),$$

and the same is true for the sample Sorensen-Dice dissimilarity:

$$d_S(\hat{\pi}) = 1 - \frac{2\hat{\pi}_{11}}{2\hat{\pi}_{11}+\hat{\pi}_{10}+\hat{\pi}_{01}} = 1 - \frac{2\hat{p}_{11}}{2\hat{p}_{11}+\hat{p}_{10}+\hat{p}_{01}} = d_S(\hat{p}),$$

with $\hat{\pi}_{ij} = n_{ij}/\nu$.

From $\pi_{11} + \pi_{10} + \pi_{01} = 1$, we have $2\pi_{11} + \pi_{10} + \pi_{01} = 1 + \pi_{11}$ and the definition of the Sorensen-Dice dissimilarity can be rewritten as:

$$d_S = d_S(\pi_{11}) = \frac{1-\pi_{11}}{1+\pi_{11}}.$$

Then, under the conditional model (conditioned to the previous observation of the $\nu$ value) finding the distribution of the sample Sorensen-Dice dissimilarity becomes a one–parameter problem: The distribution of $\hat{d}_S$ depends only on the single unknown probability $\pi_{11}$. As a consequence, finding the asymptotic distribution of $\sqrt{\nu}(d_S(\hat{\pi}_{11}) - d_S(\pi_{11}))$ is straightforward. Given the asymptotic normality of $\sqrt{\nu}(\hat{\pi}_{11} - \pi_{11})$ with asymptotic variance $\omega^2 = \pi_{11}(1-\pi_{11})$ we have:

$$\sqrt{\nu}(d_S(\hat{\pi}_{11}) - d_S(\pi_{11})) \approx N(0, \omega_S^2)$$

with

$$\omega_S^2 = \pi_{11}(1-\pi_{11})[d_S^{'}(\pi_{11})]^2 = \frac{4\pi_{11}(1-\pi_{11})}{(1+\pi_{11})^4} = \frac{4\omega^2}{(1+\pi_{11})^4}$$

which can be estimated as:

$$\hat{\omega}_S^2 = \frac{4\hat{\pi}_{11}(1-\hat{\pi}_{11})}{(1+\hat{\pi}_{11})^4} = \frac{4\hat{\omega}^2}{(1+\hat{\pi}_{11})^4}.$$

Both formulations of the standard error (i.e., $\hat{\sigma}_S/\sqrt{n}$ and $\hat{\omega}_S/\sqrt{\nu}$) coincide numerically, but the conditional formulation provides a faster computational way. First, note that the previous expression for $\omega_S^2$ (or a development in terms of all three $\pi_{ij}$ parameters) conducts to the equivalent expression:

$$\widehat{\omega}_S^2 = \frac{4\hat{\pi}_{11}\left(\hat{\pi}_{01}+\hat{\pi}_{10}\right)\left(\hat{\pi}_{11}+\hat{\pi}_{01}+\hat{\pi}_{10}\right)}{\left(2\hat{\pi}_{11}+\hat{\pi}_{01}+\hat{\pi}_{10}\right)^4}.$$

But $\hat{p}_{ij}$ and $\hat{\pi}_{ij}$ only differ in a multiplicative constant, $c = n/\nu = (n_{11}+n_{01}+n_{10}+n_{00})/(n_{11}+n_{01}+n_{10})$, so

$$\begin{aligned}
\frac{\widehat{\omega}_S(\widehat{\pi}_{11},\widehat{\pi}_{10},\widehat{\pi}_{01})}{\sqrt{\nu}} &= \frac{1}{\sqrt{\nu}}\sqrt{\frac{4\widehat{\pi}_{11}(\widehat{\pi}_{10}+\widehat{\pi}_{01})(\widehat{\pi}_{11}+\widehat{\pi}_{10}+\widehat{\pi}_{01})}{(2\widehat{\pi}_{11}+\widehat{\pi}_{10}+\widehat{\pi}_{01})^4}} \\
&= \underbrace{\sqrt{\frac{n}{\nu}}}_{\sqrt{c}}\frac{1}{n}\sqrt{\frac{4\widehat{p}_{11}(\widehat{p}_{10}+\widehat{p}_{01})(\widehat{p}_{11}+\widehat{p}_{10}+\widehat{p}_{01})c^3}{(2\widehat{p}_{11}+\widehat{p}_{10}+\widehat{p}_{01})^4c^4}} \\
&= \frac{1}{n}\sqrt{\frac{4\widehat{p}_{11}(\widehat{p}_{10}+\widehat{p}_{01})(\widehat{p}_{11}+\widehat{p}_{10}+\widehat{p}_{01})}{(2\widehat{p}_{11}+\widehat{p}_{10}+\widehat{p}_{01})^4}} \\
&= \frac{\widehat{\omega}_S(\widehat{p}_{11},\widehat{p}_{10},\widehat{p}_{01})}{\sqrt{n}}
\end{aligned}$$

It is worth to clarify that both standard error expressions can be used indistinctly but the underlying model is not the same. In all this paper we are considering the (in our opinion, more natural) unconditional model based on the four multinomial parameters $p_{11}, p_{01}, p_{10}, p_{00}$ (or three parameters $p_{11}, p_{01}, p_{10}$ –or even only two parameters $p_{11}, p_{01}+p_{10}$ for the specific case of the Sorensen-Dice dissimilarity). The above considerations on the possibility of using simplified expressions based on only one parameter, $\pi_{11}$, exclusively provide a simplified and faster way of performing some computations.
