# Supplementary material for: An equivalence test between features lists, based on the Sorensen–Dice index and the joint frequencies of GO term enrichment
Source: BMC Bioinformatics. 2022 May 31;23:207. doi: 10.1186/s12859-022-04739-2 (PMC9158181; doi:10.1186/s12859-022-04739-2)
Supplement: Supplementary file 2 — Additional file 2: goSorensen results for allOnco and PBTs lists. [file 12859_2022_4739_MOESM2_ESM.pdf]

1. AllOnco cancer data

Ontology	d_0	Level	List 1	List 2	p_{11}	p_{10}	p_{01}	p_{00}	Asymptotic normal p-value	Bootstrap p-value	n Bootstrap
BP	0,4444	03	sanger	atlas	37	0	29	447	0,00826823	0,04479552	10000
BP	0,4444	03	sanger	miscellaneous	28	9	8	468	0,001117645	0,03449655	10000
BP	0,4444	03	vogelstein	atlas	40	1	26	446	0,000446233	0,0179982	10000
BP	0,4444	03	vogelstein	miscellaneous	30	11	6	466	0,000215317	0,0179982	10000
BP	0,4444	03	vogelstein	sanger	37	4	0	472	0	0,00213458	9837
BP	0,4444	03	waldman	atlas	44	9	22	438	0,000446233	0,01329867	10000
BP	0,4444	03	waldman	miscellaneous	36	17	0	460	0	0,0059994	10000
BP	0,4444	03	waldman	sanger	33	20	4	456	0,005478965		
BP	0,4444	03	waldman	Vogelstein	35	18	6	454	0,001486348	0,03359664	10000
BP	0,4444	04	miscellaneous	atlas	189	26	197	3355	0,009668647	0,01689831	10000
BP	0,4444	04	sanger	atlas	202	25	184	3356	0,00002498	0,00209979	10000
BP	0,4444	04	sanger	miscellaneous	147	80	68	3472	0,000171854	0,00209979	10000
BP	0,4444	04	Vogelstein	atlas	221	39	165	3342	0	0,00209979	10000
BP	0,4444	04	Vogelstein	miscellaneous	150	110	65	3442	0,02022259	0,03839616	10000
BP	0,4444	04	Vogelstein	sanger	214	46	13	3494	0	0,00209979	10000
BP	0,4444	04	waldman	atlas	248	67	138	3314	0	0,00209979	10000
BP	0,4444	04	waldman	miscellaneous	203	112	12	3440	0	0,00209979	10000
BP	0,4444	04	waldman	sanger	178	137	49	3403	0,000140821	0,00209979	10000
BP	0,4444	04	waldman	Vogelstein	193	122	67	3385	0	0,00209979	10000
BP	0,4444	05	miscellaneous	atlas	453	54	485	9296	0	0,00209979	10000
BP	0,4444	05	sanger	atlas	489	59	449	9291	0	0,00209979	10000
BP	0,4444	05	sanger	miscellaneous	352	196	155	9585	0,000181745	0,00209979	10000
BP	0,4444	05	Vogelstein	atlas	529	78	409	9272	0	0,00209979	10000
BP	0,4444	05	Vogelstein	miscellaneous	365	242	142	9539	0,01025077	0,00209979	10000
BP	0,4444	05	Vogelstein	sanger	514	93	34	9647	0	0,00209979	10000

Ontology	d_0	Level	List 1	List 2	p_{11}	p_{10}	p_{01}	p_{00}	Asymptotic normal p-value	Bootstrap p-value	n Bootstrap
BP	0,4444	05	waldman	atlas	616	139	322	9211	0	0,00209979	10000
BP	0,4444	05	waldman	miscellaneous	470	285	37	9496	0	0,00209979	10000
BP	0,4444	05	waldman	sanger	436	319	112	9421	0	0,00209979	10000
BP	0,4444	05	waldman	Vogelstein	470	285	137	9396	0	0,00209979	10000
BP	0,4444	06	miscellaneous	atlas	615	75	747	15244	0,003691337	0,0059994	10000
BP	0,4444	06	sanger	atlas	674	74	688	15245	0	0,00209979	10000
BP	0,4444	06	sanger	miscellaneous	459	289	231	15702	0	0,00209979	10000
BP	0,4444	06	Vogelstein	atlas	739	100	623	15219	0	0,00209979	10000
BP	0,4444	06	Vogelstein	miscellaneous	481	358	209	15633	0	0,00209979	10000
BP	0,4444	06	Vogelstein	sanger	693	146	55	15787	0	0,00209979	10000
BP	0,4444	06	waldman	atlas	832	185	530	15134	0	0,00209979	10000
BP	0,4444	06	waldman	miscellaneous	616	401	74	15590	0	0,00209979	10000
BP	0,4444	06	waldman	sanger	563	454	185	15479	0,005126769	0,00209979	10000
BP	0,4444	06	waldman	Vogelstein	608	409	231	15433	0	0,00209979	10000
BP	0,4444	07	sanger	atlas	695	89	722	17949	0	0,00209979	10000
BP	0,4444	07	sanger	miscellaneous	452	332	226	18445	0,000185698	0,00209979	10000
BP	0,4444	07	Vogelstein	atlas	776	115	641	17923	0	0,00209979	10000
BP	0,4444	07	Vogelstein	miscellaneous	480	411	198	18366	0,000672927	0,00259974	10000
BP	0,4444	07	Vogelstein	sanger	739	152	45	18519	0	0,00209979	10000
BP	0,4444	07	waldman	atlas	815	201	602	17837	0	0,00209979	10000
BP	0,4444	07	waldman	miscellaneous	593	423	85	18354	0	0,00209979	10000
BP	0,4444	07	waldman	sanger	564	452	220	18219	0	0,00209979	10000
BP	0,4444	07	waldman	Vogelstein	612	404	279	18160	0	0,00209979	10000
BP	0,4444	08	sanger	atlas	629	90	605	17678	0	0,00209979	10000
BP	0,4444	08	Vogelstein	atlas	694	118	540	17650		0,00209979	10000
BP	0,4444	08	Vogelstein	sanger	675	137	44	18146	0	0,00209979	10000
BP	0,4444	08	waldman	atlas	692	195	542	17573	0	0,00209979	10000

Ontology	d_0	Level	List 1	List 2	p_{11}	p_{10}	p_{01}	p_{00}	Asymptotic normal p-value	Bootstrap p-value	n Bootstrap
BP	0,4444	08	waldman	miscellaneous	502	385	79	18036	0	0,00209979	10000
BP	0,4444	08	waldman	sanger	488	399	231	17884	0,002150401	0,0119988	10000
BP	0,4444	08	waldman	Vogelstein	528	359	284	17831	0	0,00209979	10000
BP	0,4444	09	sanger	atlas	485	75	462	15603	0	0,00209979	10000
BP	0,4444	09	Vogelstein	atlas	535	105	412	15573	0	0,00209979	10000
BP	0,4444	09	Vogelstein	sanger	529	111	31	15954	0	0,00209979	10000
BP	0,4444	09	waldman	atlas	519	156	428	15522	0	0,00209979	10000
BP	0,4444	09	waldman	miscellaneous	380	295	64	15886	0	0,00209979	10000
BP	0,4444	09	waldman	Vogelstein	397	278	243	15707	0,01984861	0,0319968	10000
BP	0,4444	10	sanger	atlas	328	54	310	12433	0	0,00209979	10000
BP	0,4444	10	Vogelstein	atlas	360	77	278	12410	0	0,00209979	10000
BP	0,4444	10	Vogelstein	sanger	361	76	21	12667	0	0,00209979	10000
BP	0,4444	10	waldman	atlas	341	105	297	12382	0,000157735	0,00209979	10000
BP	0,4444	10	waldman	miscellaneous	243	203	46	12633	0	0,00209979	10000
BP	0,2857	03	Vogelstein	sanger	37	4	0	472	0	0,00213849	9819
BP	0,2857	04	Vogelstein	sanger	214	46	13	3494	0	0,00209979	10000
BP	0,2857	05	Vogelstein	sanger	514	93	34	9647	0	0,00209979	10000
BP	0,2857	06	Vogelstein	sanger	693	146	55	15787	0	0,00209979	10000
BP	0,2857	07	Vogelstein	sanger	739	152	45	18519	0	0,00209979	10000
BP	0,2857	08	Vogelstein	sanger	675	137	44	18146	0	0,00209979	10000
BP	0,2857	09	Vogelstein	sanger	529	111	31	15954	0	0,00209979	10000
BP	0,2857	10	Vogelstein	sanger	361	76	21	12667	0	0,00209979	10000
CC	0,4444	03	Vogelstein	sanger	9	0	2	779	0	0,00242915	8644
CC	0,4444	04	Vogelstein	sanger	17	1	2	1417	0	0,0022096	9503
CC	0,4444	05	Vogelstein	sanger	13	0	3	2163	0	0,0022082	9509
CC	0,4444	06	Vogelstein	sanger	13	1	0	2250	0	0,00333175	6302
CC	0,4444	07	Vogelstein	sanger	15	2	1	2008	0	0,00221286	9489

Ontology	d_0	Level	List 1	List 2	p_{11}	p_{10}	p_{01}	p_{00}	Asymptotic normal p-value	Bootstrap p-value	n Bootstrap
CC	0,4444	08	Vogelstein	sanger	20	1	1	1755	0	0,00243704	8616
CC	0,4444	09	Vogelstein	sanger	9	0	2	1328	0	0,00242915	8644
CC	0,2857	03	Vogelstein	sanger	9	0	2	779		0,01452115	8676
CC	0,2857	04	Vogelstein	sanger	17	1	2	1417	0,000200915	0,00442012	9501
CC	0,2857	05	Vogelstein	sanger	13	0	3	2163	0,03119347		
CC	0,2857	06	Vogelstein	sanger	13	1	0	2250	0	0,00327715	6407
CC	0,2857	07	Vogelstein	sanger	15	2	1	2008	0,003069937	0,0154428	9518
CC	0,2857	08	Vogelstein	sanger	20	1	1	1755	0	0,00243591	8620
CC	0,2857	09	Vogelstein	sanger	9	0	2	1328		0,03631543	8673
MF	0,4444	04	Vogelstein	sanger	24	4	0	860	0	0,00213741	9824
MF	0,4444	05	Vogelstein	sanger	30	6	2	2022	0	0,00419958	10000
MF	0,4444	06	Vogelstein	sanger	20	2	1	5370	0	0,00221053	9499
MF	0,4444	07	Vogelstein	sanger	18	1	1	2696	0	0,00242803	8648
MF	0,4444	08	Vogelstein	sanger	8	0	3	1687	0,02524444		
MF	0,4444	09	Vogelstein	sanger	10	0	1	878	0	0,00332173	6321
MF	0,2857	04	Vogelstein	sanger	24	4	0	860	0	0,00427786	9817
MF	0,2857	05	Vogelstein	sanger	30	6	2	2022	0,000624255		
MF	0,2857	06	Vogelstein	sanger	20	2	1	5370	0	0,00220264	9533
MF	0,2857	07	Vogelstein	sanger	18	1	1	2696	0	0,00242971	8642
MF	0,2857	09	Vogelstein	sanger	10	0	1	878	0	0,00328896	6384

2. Kidney rejection PBTs data

Ontology	d_0	Level	List 1	List 2	p_{11}	p_{10}	p_{01}	p_{00}	Asymptotic p-value	Bootstrap p-value	n Bootstrap
BP	0,4444	03	Rej_RATs	ABMR_RATs	10	3	0	500	0,002424904		
BP	0,4444	04	Rej_RATs	ABMR_RATs	76	16	11	3664	0	0,00909909	10000
BP	0,4444	04	Rej_RATs	GRIT3	61	31	10	3665	0,19986	0,0179982	10000
BP	0,4444	05	GRIT3	GRIT2	82	46	28	10132	0,004705732	0,01779822	10000
BP	0,4444	05	LT3	LT1	17	0	7	10264	0,001115614		
BP	0,4444	05	Rej_RATs	ABMR_RATs	143	21	34	10090	0	0,00909909	10000
BP	0,4444	05	Rej_RATs	GRIT3	101	63	27	10097	0,000496253	0,00909909	10000
BP	0,4444	06	LT3	LT1	20	1	5	16655	0		
BP	0,4444	06	Rej_RATs	ABMR_RATs	191	50	55	16385	0	0,00909909	10000
BP	0,4444	06	Rej_RATs	GRIT3	145	96	34	16406	0	0,00909909	10000
BP	0,4444	07	LT3	LT1	19	1	7	19428	0,000523515		
BP	0,4444	07	Rej_RATs	ABMR_RATs	186	66	56	19147	0	0,00909909	10000
BP	0,4444	07	Rej_RATs	GRIT3	139	113	35	19168	0,01326505	0,0359964	10000
BP	0,4444	08	LT3	LT1	17	0	6	18979	0		
BP	0,4444	08	Rej_RATs	ABMR_RATs	150	62	49	18741	0	0,00909909	10000
BP	0,4444	09	Rej_RATs	ABMR_RATs	98	49	31	16447	0	0,00909909	10000
BP	0,4444	10	LT3	LT1	8	0	2	13115	0,002330402	0,01054828	8626
BP	0,4444	10	Rej_RATs	ABMR_RATs	59	34	13	13019	0,002996326	0,0359964	10000
BP	0,2857	04	Rej_RATs	ABMR_RATs	76	16	11	3664	0,000118332	0,03639636	10000
BP	0,2857	05	Rej_RATs	ABMR_RATs	143	21	34	10090	0	0,00909909	10000
BP	0,2857	06	Rej_RATs	ABMR_RATs	191	50	55	16385	0,02931452		
CC	0,4444	04	LT3	LT1	8	0	1	1428	0	0,01443299	6304
CC	0,4444	05	LT3	LT1	11	1	2	2165	0,000246679	0,01900137	9472
CC	0,4444	05	Rej_RATs	GRIT3	17	1	3	2158	0,01157676	0,00928003	9805
CC	0,4444	06	GRIT3	ABMR_RATs	26	4	0	2234	0	0,0091623	9827
CC	0,4444	06	Rej_RATs	ABMR_RATs	25	4	1	2234	0	0,0091623	9931

Ontology	d_0	Level	List 1	List 2	p_{11}	p_{10}	p_{01}	p_{00}	Asymptotic p-value	Bootstrap p-value	n Bootstrap
CC	0,4444	06	Rej_RATs	GRIT3	28	1	2	2233	0	0,00939413	9473
CC	0,4444	07	GRIT3	ABMR_RATs	24	3	0	1999	0	0,00947368	9499
CC	0,4444	07	Rej_RATs	ABMR_RATs	23	3	1	1999	0	0,00924515	9842
CC	0,4444	07	Rej_RATs	GRIT3	26	0	1	1999	0	0,01408228	6319
CC	0,4444	08	GRIT3	ABMR_RATs	21	1	0	1755	0	0,01440101	6285
CC	0,4444	08	Rej_RATs	ABMR_RATs	21	1	0	1755	0	0,01440101	6318
CC	0,4444	09	GRIT3	ABMR_RATs	18	1	0	1320	0	0,01422407	6256
CC	0,4444	09	Rej_RATs	ABMR_RATs	17	1	1	1320	0	0,01051658	8652
CC	0,4444	09	Rej_RATs	GRIT3	18	0	1	1320	0	0,01413317	6367
CC	0,4444	10	IRITD5	IRITD3	4	1	0	850		0,01469164	6193
CC	0,2857	04	LT3	LT1	8	0	1	1428	0,0122681	0,01440101	6318
CC	0,2857	05	Rej_RATs	GRIT3	17	1	3	2158	0,03487338		
CC	0,2857	06	GRIT3	ABMR_RATs	26	4	0	2234	0,1500177	0,00926209	9824
CC	0,2857	06	Rej_RATs	ABMR_RATs	25	4	1	2234	0		
CC	0,2857	06	Rej_RATs	GRIT3	28	1	2	2233	0	0,00948967	9483
CC	0,2857	07	GRIT3	ABMR_RATs	24	3	0	1999	0	0,00959005	9488
CC	0,2857	07	Rej_RATs	ABMR_RATs	23	3	1	1999	0	0,01814475	9809
CC	0,2857	07	Rej_RATs	GRIT3	26	0	1	1999	0	0,01422925	6324
CC	0,2857	08	GRIT3	ABMR_RATs	21	1	0	1755		0,01437826	6265
CC	0,2857	08	Rej_RATs	ABMR_RATs	21	1	0	1755	0	0,01437826	6328
CC	0,2857	09	GRIT3	ABMR_RATs	18	1	0	1320	0	0,0142218	6257
CC	0,2857	09	Rej_RATs	ABMR_RATs	17	1	1	1320	0	0,01045977	8699
CC	0,2857	09	Rej_RATs	GRIT3	18	0	1	1320	0	0,01407349	6394
MF	0,4444	05	Rej_RATs	ABMR_RATs	7	2	0	2051	0,02702655	0,02100404	8664
